# A Molecular Web: Endoplasmic Reticulum Stress, Inflammation, and Oxidative Stress

**DOI:** 10.3389/fncel.2014.00213

**Published:** 2014-07-29

**Authors:** Namrata Chaudhari, Priti Talwar, Avinash Parimisetty, Christian Lefebvre d’Hellencourt, Palaniyandi Ravanan

**Affiliations:** ^1^Apoptosis and Cell Death Research Lab, School of Biosciences and Technology, Vellore Institute of Technology University, Vellore, India; ^2^Groupe d’Etude sur l’Inflammation Chronique et l’Obésité, EA 41516, Plateforme CYROI, Université de La Réunion, Saint Denis de La Réunion, France

**Keywords:** endoplasmic reticulum stress, inflammation, oxidative stress, NF-κB, IRE1α, calcium

## Abstract

Execution of fundamental cellular functions demands regulated protein folding homeostasis. Endoplasmic reticulum (ER) is an active organelle existing to implement this function by folding and modifying secretory and membrane proteins. Loss of protein folding homeostasis is central to various diseases and budding evidences suggest ER stress as being a major contributor in the development or pathology of a diseased state besides other cellular stresses. The trigger for diseases may be diverse but, inflammation and/or ER stress may be basic mechanisms increasing the severity or complicating the condition of the disease. Chronic ER stress and activation of the unfolded-protein response (UPR) through endogenous or exogenous insults may result in impaired calcium and redox homeostasis, oxidative stress via protein overload thereby also influencing vital mitochondrial functions. Calcium released from the ER augments the production of mitochondrial Reactive Oxygen Species (ROS). Toxic accumulation of ROS within ER and mitochondria disturbs fundamental organelle functions. Sustained ER stress is known to potentially elicit inflammatory responses via UPR pathways. Additionally, ROS generated through inflammation or mitochondrial dysfunction could accelerate ER malfunction. Dysfunctional UPR pathways have been associated with a wide range of diseases including several neurodegenerative diseases, stroke, metabolic disorders, cancer, inflammatory disease, diabetes mellitus, cardiovascular disease, and others. In this review, we have discussed the UPR signaling pathways, and networking between ER stress-induced inflammatory pathways, oxidative stress, and mitochondrial signaling events, which further induce or exacerbate ER stress.

## Introduction

Endoplasmic reticulum (ER) is a cardinal membrane-bound organelle comprising of interconnected highly branched tubules, vesicles, and cisternae. ER structure can be categorized as domains like nuclear envelope domain (array of proteins that are synthesized on rough ER and concentrated within the inner membrane), the rough and smooth ER domain (due to presence and absence of bound ribosomes respectively), and the regions that contact other organelles like plasma membrane, Golgi, vacuoles, mitochondria, peroxisomes, late endosomes, and lysosomes (for ease of direct transfer of lipids to their membranes and efficient calcium signaling) (Voeltz et al., 2002). ER functions majorly in translocating and integrating proteins (secretory and membrane proteins respectively), assisting their folding and transport (extracellular or to cell membrane), lipid biosynthesis, and maintaining calcium homeostasis. It is also a site for post translational modification (*N*-linked glycosylation) of proteins and is considered as a signaling organelle (Berridge, 2002; Fagone and Jackowski, 2009; Sammels et al., 2010; Braakman and Bulleid, 2011). Ribosomes embedded on rough endoplasmic reticulum (RER) are sites for protein synthesis and secretion. Smooth endoplasmic reticulum (SER) lacks bound ribosomes, therefore, inefficient in protein synthesis but is important for fatty acid and phospholipids synthesis, carbohydrate metabolism, lipid bilayer assembly, and regulation of calcium homeostasis. Cell type, cell function, and cell needs determine the role of ER in the cell. For example, liver cells high in SER aid in drug detoxification; while plasma cells, beta cells, and other secretory cells are rich in RER to meet their secretory protein demand; sarcoplasmic reticulum, a specialized form of the ER is more in muscle cells functioning in muscle contractions and relaxation (Alberts et al., 2002).

The excursion of secretory or membrane protein begins at ER. A protein destined to be modified in ER is marked with an N-terminal ER signal sequence. During co-translational modification, a signal recognition particle (SRP) recognizes and binds to signal sequence on nascent protein, ribosome, and SRP receptor on ER membrane, after which ribosome-nascent polypeptide chain complex is rapidly transferred to a membrane protein translocator, the SEC61 translocon. Signal peptide is cleaved by signal peptidase on ER membrane and finally the nascent chain enters the ER lumen via the translocon. In post translational modification, interaction of SEC61 translocon and completely synthesized protein is sufficient for its ER targeting (Lodish et al., 2000).

The proteins in ER are folded and modified under the vigilance of ER resident molecular chaperones and folding enzymes that accelerate rate limiting reactions in protein folding mechanisms and thus assist them in attaining appropriate conformation and maturation. The glucose-regulated proteins (GRP) system including chaperones of Hsp70 family and co-chaperones of HSP40 family (Kampinga and Craig, 2010), ER lectin-like chaperone system including calnexin (CNX) and/or calreticulin (CRT) along with glucosidases and transferases (Rutkevich and Williams, 2011), and finally the protein disulfide isomerase (PDI) family of disulfide bond oxidase, reductase, and isomerase enzymes (Appenzeller-Herzog and Ellgaard, 2008) are the pillars of Endoplasmic Reticulum Quality Control system (ERQC). ERQC system has its particular vital role to play in converting a protein from nascent to native state (Nishikawa et al., 2005; Bukau et al., 2006; Hebert and Molinari, 2007; Araki and Nagata, 2011). The aptly folded proteins are transported to Golgi via vesicular carriers and finally escorted to their destinations (plasma membrane or lysosomal membrane or loaded into granules for secretion) (Szul and Sztul, 2011). Selective chaperones of ERQC system and specific mannose lectins like ER degradation-enhancing α-mannosidase-like protein (EDEM) are capable of tagging unassembled, misfolded, or unfolded proteins, which facilitates their identification and retrotranslocation to cytosol via SEC61 translocon where they are steered to ubiquitin proteasome degradation system or are eliminated by autophagic degradation (Smith et al., 2011).

Here, we review the signaling events occurring within ER under stressed conditions, its counter effect on other organelles, and give crisp information on interaction between ER stress pathways, oxidative stress, and inflammation.

## Unfolded-Protein Response

Accumulation of unfolded/misfolded/mutated proteins (Hetz and Soto, 2006; Viana et al., 2012), disturbances in cellular redox regulation and endogenous reactive oxygen species (ROS) production (Fedoroff, 2006), hypoxia (Feldman et al., 2005; Sawada et al., 2008), hyperglycemia, and hyperlipidemia (Fonseca et al., 2011; Back et al., 2012), aberrations in calcium regulation (Gorlach et al., 2006), viral infections (He, 2006; Zhang and Wang, 2012) act as stress signals and alter ER homeostasis making it dysfunctional. In response to such diverse signals, ER elicits a protective or adaptive response called unfolded-protein response (UPR) with an aim to restore ER homeostasis; however, if the stress signal is severe and/or prolonged, ER triggers cell death pathways (Szegezdi et al., 2006; Kim et al., 2008a; Cheng and Yang, 2011; Benbrook and Long, 2012).

Stress signals culminate in overloading ER with proteins and exhausting the ER machinery. ER stress is thought to be and in certain cases proved to play a key role in diseases like Alzheimer’s disease (Salminen et al., 2009; Viana et al., 2012), Parkinson’s disease (Wang and Takahashi, 2007; Cali et al., 2011), amyotrophic lateral sclerosis (ALS) (Lautenschlaeger et al., 2012; Tadic et al., 2014), poly glutamine diseases (Vidal et al., 2011), ischemia (Doroudgar et al., 2009), atherosclerosis (Zhou and Tabas, 2013), bipolar disorder (Hayashi et al., 2009), prion diseases (Xu and Zhu, 2012), cancer (Tsai and Weissman, 2010), diabetes (Papa, 2012), auto immune disorders (Zhong et al., 2012), and cardiovascular disorders (Minamino et al., 2010). Interestingly, there are reports demonstrating that ER stress inhibition could protect against neuronal injury (Qi et al., 2004; Sokka et al., 2007), ischemia (Nakka et al., 2010), cardiovascular diseases (Teng et al., 2011), respiratory disorders (Hoffman et al., 2013), atherosclerosis (Zhou et al., 2013), and sleep apnea (Zhu et al., 2008), in *in vivo* murine models.

## The Machinery of UPR

**Figure 1:**
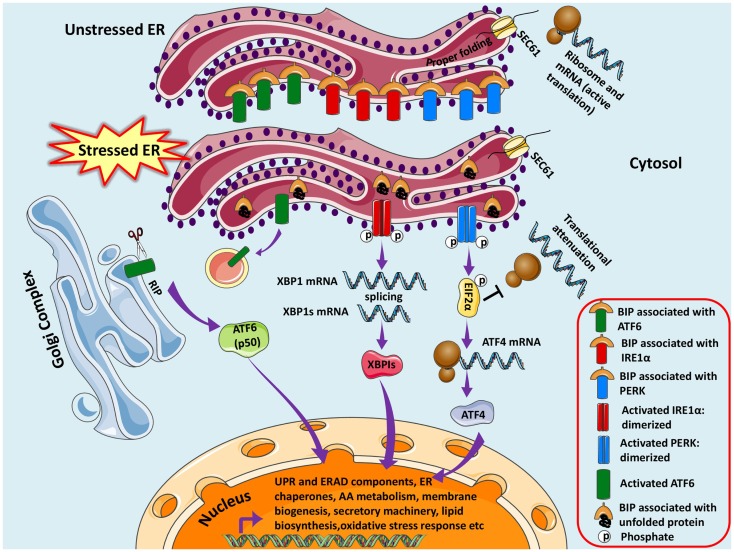
**Unfolded protein response pathways are shown**. The newly synthesized protein destined for modification in ER enters through SEC61 channel and undergoes folding and maturation. Under basal/unstressed conditions, the BIP (immunoglobulin heavy chain binding protein) binds to ATF6 (activating transcription factor 6), IRE1α (inositol-requiring kinase 1), and PERK [double-stranded RNA-activated protein kinase (PKR)-like endoplasmic reticulum kinase] thereby inhibiting them. During ER stress, BIP dissociates from the three UPR sensors and binds to unfolded/misfolded proteins, thus initiating adaptive signaling events to help ER recover from the stress. Dissociation of BIP from ATF6 unmasks a Golgi-localization signal (GLS, not shown here), which facilitates its translocation to Golgi where it undergoes regulated intramembrane proteolysis (RIP) by resident proteases, Site-1 protease (S1P) and Site-2 protease (S2P). The released ATF6 acts as a transcription factor, which travels to the nucleus and binds to ER-stress response elements (ERSE) and induces transcription of several genes, including BIP, CHOP (CCAAT/enhancer-binding protein homologous protein), and X-box-binding protein 1 (XBP1). Similarly IRE1α is activated and undergoes homodimerization and autophosphorylation, thereby activating the endoribonuclease activity, which splices XBP1 mRNA to spliced XBP1 mRNA, which codes for a transcription factor XBP1s that translocates to nucleus and regulates genes involved in UPR and ER-associated degradation (ERAD). Finally, the release of BIP activates PERK pathway, which initiates a global translational arrest by phosphorylating the translation initiation factor 2α (EIF2α), thus decreasing ER protein load. ATF4 (Activating Transcription Factor 4) mRNA escapes translational suppression exclusively as it possesses internal ribosome entry site (IRES) sequences in the 5′-untranslated regions. ATF4 enters nucleus and regulates expression of UPR target genes.

The adaptive UPR comprises of signal transduction pathways initiated by ER proximal UPR transmembrane proteins: inositol-requiring kinase 1 (IRE1α), activating transcription factor 6 (ATF6), and double-stranded RNA-activated protein kinase (PKR)-like endoplasmic reticulum kinase (PERK) in an attempt to restore homeostasis and normal ER functions (Schroder and Kaufman, 2005). These UPR transducer proteins are negatively regulated by the chaperone GRP78/BIP (immunoglobulin heavy chain binding protein) in unstressed or healthy ER at their luminal ends (amino terminal), however, increase in unfolded proteins causes dissociation of Grp78/BIP thereby releasing the inhibition and thus eliciting the response (Bertolotti et al., 2000; Pfaffenbach and Lee, 2011) (Figure 1).

### The IRE1α pathway

Homo-oligomerization of activated IRE1α opens the Ser/Thr kinase domain at the cytosolic carboxyl terminal, aligning it for trans-autophosphorylation thereby activating the endoribonuclease domain (Shamu and Walter, 1996; Sidrauski and Walter, 1997; Liu et al., 2003). X-box-binding protein 1 (XBP1) mRNA is spliced unconventionally by this RNAase domain of IRE1α; cleaving a 26 nucleotide intron to produce a spliced mRNA that codes for bZIP-family transcription factor sXBP1 (spliced XBP1). Once it is translocated to the nucleus, it can dimerize or act in tandem with other co-regulators and regulate several genes involved in UPR and ER-associated degradation (ERAD) by binding to ER stress response element (ERSE) promoter (Yoshida et al., 1998, 2001a; Lee et al., 2003; Van Huizen et al., 2003). The dimerized and activated IRE1α collaborates with modulators and adaptors on the cytosolic end to initiate signaling events in response to the intensity and duration of stress. Adaptor-like tumor necrosis factor receptor (TNFR)-associated factor 2 (TRAF2), an E3 ubiquitin ligase, recruits apoptosis signal-regulating kinase (ASK1), a mitogen-activated protein kinase kinase kinase (MAPKKK) that has been shown to relay various stress signals to the downstream MAPKs consequently activating Jun N-terminal kinase (JNK) and p38 MAP kinase (Derijard et al., 1995; Urano et al., 2000; Nishitoh et al., 2002). IRE1α also triggers activation of other kinases such as extracellular signal-regulated kinases (ERKs) as well as nuclear factor κB (NF-κB) pathways (Kaneko et al., 2003; Nguyen et al., 2004; Hu et al., 2006).

### The PERK pathway

Double-stranded RNA-activated protein kinase (PKR)-like endoplasmic reticulum kinase, another UPR mediator, also undergoes similar dimerization and autophosphorylation (Ma et al., 2002a) to initiate a transient cellular translational arrest by phosphorylating at serine residue 51 and inactivating eukaryotic translation initiation factor (eIF2α) (Harding et al., 1999), the most studied and well-known substrate of PERK, thereby reducing protein load on ER and causing cell-cycle arrest (Brewer and Diehl, 2000). However, the phosphorylated eIF2 α can translate genes with internal ribosome entry site (IRES) sequences in the 5′-untranslated regions sidestepping the eIF2α translational block (Vattem and Wek, 2004; Schroder and Kaufman, 2005); one such gene being *ATF4*, which encodes ATF4 (Activating Transcription Factor 4), a member of the cAMP response-element-binding (CREB) family of transcription factors (Lu et al., 2004) that induces expression of several genes central in cell survival and UPR (Harding et al., 2000a,b, 2003; Scheuner et al., 2001; Ma et al., 2002b; Raven et al., 2008). This PERK-mediated translational block is also essential for activation of NF-κB (Jiang et al., 2003). One of the downstream substrate of PERK is Keap1, which sequesters the nuclear erythroid 2 p45-related factor 2 (Nrf2) in cytoplasm. PERK phosphorylates Keap1, thereby freeing Nrf2 of inhibition and facilitating its nuclear import for expression of antioxidant and detoxification enzymes (Cullinan et al., 2003; Cullinan and Diehl, 2006).

### The ATF6 pathway

Release of GRP78, unmasks the Golgi-localization signal (GLS), forms ER membrane tethered ATF6 proteins expediting their translocation to the Golgi (Chen et al., 2002); where they undergo regulated intramembrane proteolysis (RIP) by resident proteases Site-1 protease (S1P) and Site-2 protease (S2P) (Ye et al., 2000) cleaving at a juxtamembrane site, producing and releasing active ATF6 transcription factors into the cytosol (Haze et al., 1999). They will then migrate into the nucleus and homodimerize or heterodimerize with other transcription factors to regulate gene expression of proteins involved in UPR (Yoshida et al., 1998, 2001b; Yamamoto et al., 2007; Adachi et al., 2008). Not a long ago, new membrane-bound bZIP transcription factors were identified with similar structural and proteolysis pattern as ATF6. These include Luman (CREB3), Oasis (old astrocyte specifically induced substance, CREB3L1), BBF2H7 (CREB3L2), CREBH (CREB3L3), and Tisp40 (CREB4, CREB3L4) (Bailey and O’Hare, 2007). Regardless of structural similarity with ATF6, these factors execute distinctive functions in regulating UPR based on activating stimuli/stress, cell type, and response element binding (Asada et al., 2011). CREBH, a liver-specific transcription factor does not serve as a UPR transactivator for expression of classic ER chaperone genes regulated under ERSE; however, it modulates ER stress response genes that contain ERSE in their promoter regions. CREBH is cleaved upon ER stress thereby activating expression of acute phase response (APR) genes like those encoding serum amyloid P-component (SAP) and C-reactive protein (CRP) providing a correlation between ER stress and acute inflammatory responses (Zhang et al., 2006).

Thus, all the three UPR sensors are regulated by one master regulator, i.e., GRP78/BIP (Hendershot, 2004). The mission of ER under stress is to thus increase the expression of transcription factors, which orchestrates and initiates production of ER chaperones and genes involved in ERAD, to provide tolerance to the stress and restore homeostasis. Thus, ER mandatorily has to be dynamic to meet its changing needs, which are managed by integrated signaling pathways that constantly monitor the levels of ER machinery. If the UPR is incompetent to abolish the stress, ER prompts a cell death program, which can be apoptotic (Szegezdi et al., 2006; Tabas and Ron, 2011; Gorman et al., 2012), non-apoptotic (Ullman et al., 2008), or autophagic (Ogata et al., 2006; Yorimitsu et al., 2006; Ullman et al., 2008; Cheng and Yang, 2011) therefore resulting in cellular demise (Heath-Engel et al., 2008; Benbrook and Long, 2012; Logue et al., 2013).

## Integration of ER Stress and other Pathways in Neurodegeneration

**Figure 2:**
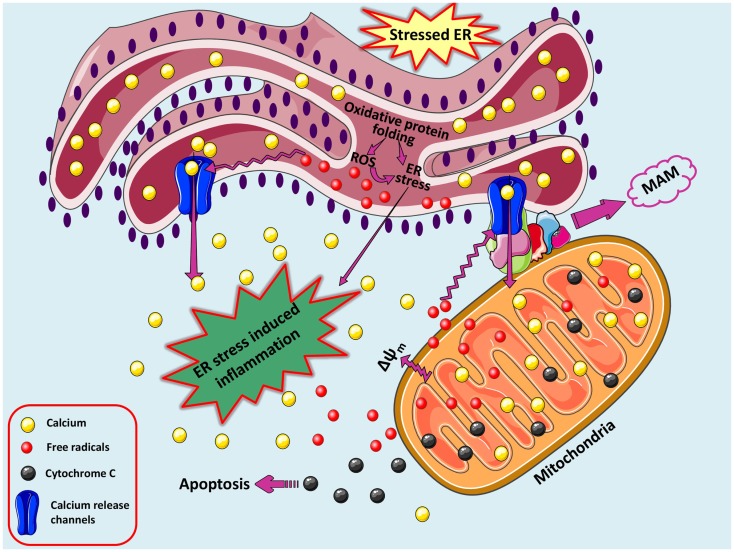
**A loop of oxidative stress, ER stress leading to inflammation**. During protein overload, ROS are generated in the ER as a part of an oxidative folding process during electron transfer between protein disulfide isomerase (PDI) and endoplasmic reticulum oxidoreductin-1 (ERO-1). ROS can target ER resident proteins, enzymes, and chaperones (not shown) and ER based calcium (Ca^2+^) channels, leading to the release of calcium from the ER into the cytosol and ER-stress signaling. Increased cytosolic calcium and calcium entry in mitochondria from ER via MAM-associated channels can stimulate mitochondria metabolism to produce more ROS. Increased mitochondrial calcium concentration causes cytochrome *c* release, altered membrane potential that eventually triggers cellular death programs. The increased protein folding demand, calcium and ROS signaling integrates with UPR pathways and can potentially lead to inflammatory responses.

The major cellular communicating compartments include nucleus, ER, mitochondria, and Golgi that initiate signaling pathways to help the cell respond to various intracellular and extracellular signals/stresses. Besides UPR, other pathways emanating from ER collaborate with mitochondria and nucleus to regulate the cellular responses (Figure 2). The interaction among reactive species formation, disturbed calcium homeostasis, mitochondrial collapse, and inflammation is a common phenomenon existing in various disorders and its connection with ER stress is recently being explored and demands more research. The complications underlying neurodegenerative disorders are multi-factorial and may include genetic predisposition (Bertram and Tanzi, 2005; Lill and Bertram, 2011), environmental factors (Cannon and Greenamyre, 2011), cellular stressors such as oxidative stress and free radical production (Gandhi and Abramov, 2012), excitotoxicity (Dong et al., 2009), neuroinflammation (Glass et al., 2010), disruption of calcium-regulating systems (Bezprozvanny, 2009), mitochondrial dysfunction (Lezi and Swerdlow, 2012), and misfolded protein accumulation (Matus et al., 2011).

Central nervous system being most aerobic includes neurons, which are differentiated and are highly mitochondrial dependent. They require massive amount of adenosine 5-triphosphate (ATP); to satisfy their tremendous metabolic demands, for maintaining ionic gradients across the cell membranes and for neurotransmission, thereby being very sensitive to mitochondrial stress (Ames, 2000; Nicholls and Budd, 2000). Mitochondria are known to play a central role in many vital cell functions including generation of cellular energy in the form of ATP, maintaining intracellular calcium homeostasis, ROS formation, apoptosis, fatty acid oxidation, and steroid synthesis. Mitochondrial stress characterized by abnormal mitochondrial morphology, biochemical dysfunction, and disturbed redox homeostasis is a common manifestation in brain aging and several chronic neurodegenerative diseases like Alzheimer’s disease, Parkinson’s disease, ALS, and Huntington’s disease (Beal, 2005; Lezi and Swerdlow, 2012). Likewise, mitochondrial dysfunction is also predominant in pathogenesis of cancer, cardiovascular diseases, and several metabolic diseases like obesity, insulin resistance, and diabetes (Lesnefsky et al., 2001; Wallace, 2005; Kim et al., 2008b; Mantena et al., 2008; Hu and Liu, 2011).

Mitochondria have a noteworthy role in neurodegenerative diseases, where various disease-specific proteins (misfolded/mutated) interact with mitochondria and/or affect mitochondrial function (Johri and Beal, 2012). There are emerging evidences suggesting that triggering of UPR pathways by cellular and/or extracellular accumulation of misfolded/aberrant proteins in several human neurodegenerative conditions could cause severe loss in neuronal functions and viability (Doyle et al., 2011; Bernales et al., 2012). In addition, imbalance in Ca^2+^ homeostasis, which is significant in neurodegenerative diseases affects mitochondria and ER as they are physically and functionally tightly interconnected via mitochondrial-associated membranes (MAM), which participates in Ca^2+^ signaling, lipid transport, energy metabolism, and cell survival. Thus, apart from coordinating pathways involved in cell life and death, their physical association is vital and any loss of communication in them is known to complicate and worsen the prevailing conditions in neurodegenerative diseases (Malhotra and Kaufman, 2011; Vannuvel et al., 2013).

### Calcium links ER and mitochondria

In the brain, calcium participates in various intracellular and extracellular processes and its fluctuations across the plasma membrane and between intracellular compartments play crucial roles in integral functions of neurons like neuronal excitability, synaptic transmission and plasticity, memory formation, neurotransmitter release, activation of specific calcium-dependent signal transduction pathways, and gene transcription (Berridge, 1998). The free cytosolic calcium levels in neurons are maintained around 0.1–0.5 μM, while the extracellular levels being ~1 mM. On encountering stimulatory signals, extracellular calcium influx or calcium release from intracellular stores is triggered. Calcium can enter the neuronal cell by voltage-operated channels or by receptor-operated channels, on the plasma membrane controlled by neurotransmitters. Later includes glutamate receptors like *N*-methyl-d-aspartate (NMDA), α-amino-3-hydroxy-5-methylisoxazole-4-propionate acid (AMPA), kainic acid, nicotinic acetylcholine (nACh), serotonin (5-HT3) and adenosine 5-triphosphate (ATP) P2X receptors. Na-K-ATPase and Ca^2+^-ATPases maintain ionic gradient along the neuronal membrane at the expense of considerable amounts of ATP. The main intracellular calcium store is the ER (10–100 mM), acting as a major buffering system that functions as a sink for Ca^2+^ storage, from where calcium can be released into the cytosol via activation of the inositol 1,4,5-triphosphate receptors [InsP3Rs, sensitive to inositol 1,4,5-triphosphate (InsP3)] or ryanodine receptors (RyRs, sensitive to cyclic ADP ribose). Both the InsP3Rs and the RYRs are also sensitive to Ca^2+^, and this process of calcium-induced calcium release can set up propagated calcium waves. Resting cytosolic calcium levels are partly maintained by calcium-binding and calcium-buffering proteins (e.g., calmodulin, calcineurin, calretinin, calbindin, or parvalbumin) or by active uptake into internal stores by the Sarco/ER calcium-ATPase (SERCA) at the ER membrane or by the mitochondrial uniporter. Minor transient alterations in calcium levels occurring during physiological processes are salient to neuronal functioning; however, pathological conditions accompanied by prolonged increase in calcium levels can overwhelm the calcium regulatory network, making the cell difficult to recuperate. Rampant fluctuations in this highly regulated calcium homeostasis not only disturbs normal brain physiology but also hampers neuronal integrity and viability (Kawamoto et al., 2012). An accumulation of misfolded proteins in the ER lumen can cause leaky calcium efflux from the ER, possibly via inositol-triphosphate receptors (Deniaud et al., 2008). Additionally during Ca^2+^ overload, calcium influx increases in mitochondria and ER, thereby causing changes in mitochondrial pH and ROS production accompanied by altered mitochondrial membrane potential and opening of permeability transition pore with subsequent release of cytochrome *c*, cardiolipin peroxidation, and activation of several calcium-dependent proteins and kinases (Smaili et al., 2009). Thus, calcium-induced ROS increase and ROS-mediated calcium vulnerability create a self amplifying loop (Peng and Jou, 2010).

In the adaptive phase of UPR, ER-to-mitochondria Ca^2+^ transfer under regulation by MAM proteome maintains mitochondrial metabolism and ATP production that safeguards cellular functions. However, severe ER stress induces mitochondrial Ca^2+^ overload, ROS accumulation, and ATP depletion and thus activates the mitochondria-dependent apoptosis (Raturi and Simmen, 2013). Prolonged stimulation of neuronal glutamate receptors results in excitotoxicity due to massive calcium influx activating several enzymes and proteases eventually causing neuronal loss in neurodegenerative diseases. This is accompanied by excessive nitric oxide (NO) production by neuronal NO synthase (Calabrese et al., 2000; Dong et al., 2009). Significant loss in PDI function due to NO-mediated S-nitrosylation causes dysregulated protein folding and accumulation of polyubiquitinated proteins leading to neuronal death via ER stress. This was backed up by the fact that *S*-nitrosylated PDI was found in neurodegenerative diseased brains suggesting ER dysfunction as critical factor that relates NO-induced cellular stress to neurodegeneration (Uehara et al., 2006). Chen et al. (2013) identified NO-mediated S-nitrosylation of PDI as one of the liable factors for accumulation of mutant superoxide dismutase (SOD) 1 aggregates in ALS.

### Oxidative stress in ER and mitochondria

Free radicals are generated from two sources: the UPR-regulated oxidative folding machinery in the ER (Bhandary et al., 2012) and mitochondria (Cadenas and Davies, 2000). ROS and reactive nitrogen species (RNS) are byproducts of normal cellular metabolism and are also produced in response to multiple stresses in various compartments of cell by enzymatic or non-enzymatic processes. ROS and RNS are known to play dichotomous role as being both harmful at high concentrations while beneficial at moderate/low concentrations and in harmony with cellular antioxidant defense mechanisms that they maintain cellular redox homeostasis (Calabrese et al., 2000; Finkel, 2011; Dasuri et al., 2013). Oxidative stress is a condition in which the production of reactive species is alarmingly high and the available antioxidant defenses is limited; resulting in damage to DNA, proteins, sugars, and lipids by the excessive free radicals (Valko et al., 2007). Superoxide anion (triplet stage molecular oxygen) produced majorly in mitochondria is a primary ROS and is a precursor for many secondary ROS, including hydrogen peroxide, hydroxyl radical, hypochlorous acid, and hydroperoxyl radical. Similarly nitric oxide is produced during arginine metabolism and can be converted to various other RNS such as nitrosonium cation, nitrite and nitrate radical, nitroxyl anion, or peroxynitrite. Complexes I and III of mitochondrial respiratory chain and α-ketoglutarate dehydrogenase in the tricarboxylic acid (TCA) cycle contribute to ROS production. There also exist mitochondrial enzymes that detoxify/scavenge free radicals (Stamler et al., 1992; Turrens, 2003).

Endoplasmic reticulum provides an exclusive oxidizing-folding environment to the proteins to facilitate disulfide bond formation and this process is believed to contribute to 25% of ROS generated by cell (Tu and Weissman, 2004; Malhotra and Kaufman, 2007). PDI, a member of the thioredoxin superfamily catalyzes disulfide bond formation through thiol-disulfide oxidation, reduction, and isomerization. A flavin adenine dinucleotide (FAD)-dependent reaction is carried out by ERO-1 (ER membrane associated oxidoreductin) involving transfer of electrons from reduced PDI to molecular oxygen (O_2_), resulting in ER protein folding-induced oxidative stress. Erroneous disulfide bonds are reduced by glutathione (GSH) thereby decreasing the reduced glutathione to oxidized glutathione (GSSH) ratio and altering the redox environment in the ER. The ER ratio of GSH/GSSH ranges from 1:1 to 3:1, while the cellular ratio ranges from 30:1 to 100:1. Lastly, ER transmembrane protein NADPH oxidase complex, Nox4 is also involved in producing superoxide anion and hydrogen peroxide (Hwang et al., 1992; Santos et al., 2009).

## ER Stress and Inflammation

Endoplasmic reticulum stress and inflammation are related in a way that under acute trigger, they safeguard the cellular viability and functions and when chronically induced they are destructive and go beyond physiological control. Recent research reveal connections at multiple levels between UPR and inflammation and therefore focuses are now deviated to understand involvement of ER stress in specialized cells and tissues concerning inflammatory and immune responses. ER stress-induced inflammation primarily serves to limit the tissue damage and facilitate tissue repair; however, it largely depends on the target cell type, the disease stage, and the type of ER stressor. Several inflammatory chronic diseases including diabetes, obesity, neurodegenerative diseases, atherosclerosis, arthritis, respiratory diseases, irritable bowel syndrome, cardiovascular diseases, cancer, and many metabolic diseases have ER stress as factor involved in progression of the disease (Pahl and Baeuerle, 1995; Zhang and Kaufman, 2008; Garg et al., 2012; Verfaillie et al., 2013).

### NF-κB – a key player in mediating inflammatory response

NF-κB plays an essential role of transcriptional regulator in mediating inflammatory responses, immune and stress responses, and regulates apoptosis, proliferation, differentiation, and development. Under various stimulatory conditions by inflammatory cytokines, viral and bacterial infections, or physical trigger by UV irradiation or cellular physiological stresses, NF-κB is activated and NF-κB target genes are expressed. NF-κB is a dimeric protein comprising of different combinations of five Rel family members [p65 (RelA), RelB, c-Rel, p105/p50 (NF-κB1) and p100/52 (NF-κB2)] therefore forming homo or heterodimers. They contain a conserved ~300 amino acid region called Rel Homology Region (RHR), which is composed of DNA-binding domain at its N-terminal, followed by a short flexible linker, C terminal dimerization domain, and finally a nuclear localization signal (NLS) that completes the RHR. p65/RelA, c-Rel, and RelB, contain a transcription activation domain (TAD) beyond the RHR at extreme carboxy terminal ends and are responsible for transcribing NF-κB target genes. Consequently, NF-κB dimers having minimum one of them (p65/RelA, c-Rel, and RelB) are active transcription factors. NF-κB dimers solely made of p50 and p52 subunits repress transcription since they lack TAD, inspite of being capable of nuclear localization and DNA binding. Unless required, NF-κB is sequestered in cytoplasm in its inactive form by constitutively expressing inhibitors of NF-κB (IκB: IκBα, IκBβ, IκBε) and is activated by signal-induced phosphorylation and consequential proteosome degradation of inhibitor by IκB kinase (IKK). IκBα, IκBβ, and IκBε contain a central ankyrin repeat domain (ARD), which has conserved serine residues for phosphorylation by IKK and also conserved lysine amino acids for poly-ubiquitination at its amino terminal. IκBα exclusively has a functional nuclear export sequence (NES), which is not masked on binding to NF-κB therefore restrains the complex NF-κB:IκBα to cytoplasm. NF-κB:IκBβ complexes can reside stably in either nucleus or cytoplasm. IκBε and IκBα are found to function as negative feedback regulators for NF-κB escorting it back to cytoplasm (Arenzana-Seisdedos et al., 1995; Kearns et al., 2006). All the three inhibitors of NF-κB at their carboxy terminal ends have a short sequence rich in the amino acids proline, glutamic acid, serine, and threonine. NF-κB precursor’s p100 and p105 are also inhibitors of NF-κB in an entire different fashion involving multiprotein assemblies. Dissociation of IκB from NF-κB unmasks NLS, which facilitates NF-κB’s translocation in nucleus thereby transcribing NF-κB target genes involved in inflammatory responses. The IKK is composed of three subunits: IKKα and IKKβ are legitimate kinases while IKKγ (NEMO, NF-kappa-B essential modulator which interacts with IKKβ) is known to play a regulatory role instead. The canonical pathway involves activation of cytokine receptors [(TNFR), interleukin 1 (IL-1) receptor (IL-1R)], antigen receptors and pattern-recognition receptors, Toll-like receptor 4 (TLR4), and subsequent phosphorylation of IκBα by IKKβ and NEMO thereby freeing mostly p65-containing heterodimers for nuclear translocation. The alternative or non-canonical pathways are triggered by activation of specific members of the TNF cytokine family, such as CD40 ligand, B cell activating factor (BAFF) and lymphotoxin-β following IKKα-mediated processing of precursor protein p100 to p52 forming active p52–RelB complexes. NF-κB-inducing kinase (NIK) is known to activate IKKα, whereas IKKβ can be activated by multiple kinases (Dixit and Mak, 2002; Hayden and Ghosh, 2008; Huxford and Ghosh, 2009; Lawrence, 2009; Oeckinghaus et al., 2011).

## The ER Stress as a Trigger for Inflammation

Pahl and Baeuerle in 1995 demonstrated that NF-κB as a mixture of p5O/p65 and p5O/c-rel heterodimers, functions in a novel ER-nucleus signal transduction pathway upon gradual accumulation of proteins in ER caused by over expression of the immunoglobulin μ heavy chains. Similarly, tunicamycin, 2-deoxyglucose, brefeldin A, and thapsigargin are capable of eliciting UPR (grp induction) and activating NF-κB. However, castanospermine (glucosidase inhibitor) and dithiothreitol (DTT) potentially activate the UPR but not the NF-κB pathway; and tumor necrosis factor α (TNFα) and p65 over expression activate NF-κB but not the UPR. On the other hand, synthetic triterpenoids such as 2-cyano-3,12-dioxoolean-1,9-dien-28-oic acid (CDDO), Methyl 2-cyano-3,12-dioxooleana-1,9(11)dien-28-oate, Bardoxolone methyl (CDDO-Me) and cyano enone of methyl boswellates (CEMB) have been known to induce UPR and block the NF-κB signaling (Zou et al., 2008; Ravanan et al., 2011a,b). Another interesting finding was that preincubation with the antioxidant pyrrolidinedithiocarbamate (PDTC) inhibited the NF-κB activation induced by tunicamycin, 2-deoxyglucose, and brefeldin A implicating oxidative stress as a trigger for NF-κB activation (Pahl and Baeuerle, 1995). In their further research, they found that ER stress-mediated NF-κB activation depends on ER calcium efflux (by thapsigargin or cyclopiazonic acid), followed by formation of reactive oxygen intermediates (ROI). Preincubation with calcium chelators like 1,2-bis(2-aminophenoxy)ethane-*N*,*N*,*N*′,*N*′-tetraacetic acid tetrakis(acetoxymethyl)ester (BAPTA-AM) and 3,4,5-trimethoxybenzoic acid 8-(diethylamino)octyl ester (TMB-8); and antioxidants like *N*-acetyl-l-cysteine (NAC), butylated hydroxyanisole (BHA), rotenone, nordihydroguaiaretic acid (NDGA), DTT, and pyrrolidine dithiocarbamate (PDTC, to less extent) blocked NF-κB activation. In addition, calcium chelators inhibited ROI formation in response to thapsigargin and cyclopiazonic acid, implying that calcium release precedes ROI formation ultimately mediating ER overload-dependent NF-κB activation. Use of tepoxalin showed that ROI production was a result of cyclooxygenases or lipoxygenases in response to thapsigargin (Pahl and Baeuerle, 1996, 1997).

**Figure 3:**
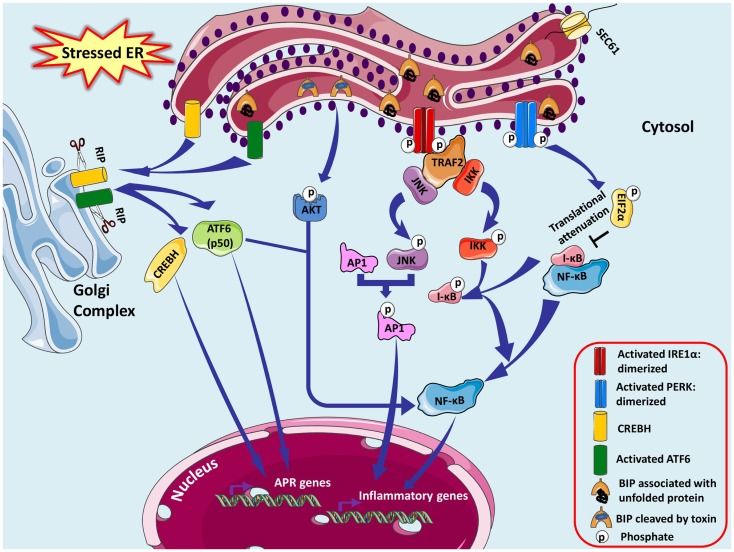
**Endoplasmic reticulum stress-associated NFκB activation is shown**. ER stress-induced activation of IRE1α kinase activity recruits adaptor protein tumor-necrosis factor-α (TNF-α) receptor-associated factor 2 (TRAF2), which further recruits c-Jun N-terminal kinase (JNK) activating several transcription factors and many apoptosis signaling proteins. JNK induces the expression of inflammatory genes by phosphorylating the transcription factor activator protein 1 (AP-1), which is a dimer of monomers from various protein families forming different combinations of AP-1. They induce inflammation via promoting transcription of genes for cytokines, chemokines, and other proinflammatory molecules. TRAF2 associates with IκB kinase (IKK) activating NF-κB by promoting degradation of IκBα resulting in NF-κB nuclear translocation. PERK branch of UPR also can potentially induce NF-κB not by IκBα phosphorylation or degradation but essentially by its eIF2α-mediated attenuation of translation; successively inhibiting the synthesis of IκBα. Subtilase cytotoxin from Shiga strain of *E. coli* makes a specific single site cleavage in GRP78, therefore capable of eliciting ER stress response pathways. CREBH and ATF6 may dimerize and synergistically activate transcription of major APR genes inducing a systemic inflammatory response in specific cells.

The close link between ER stress and inflammation is a likely contributor to the integration of ER function and metabolic homeostasis, considering the significant role of inflammation in metabolic diseases (Hotamisligil, 2006). For instance, leptin (an important adipokine acts on receptors in the hypothalamus of the brain where it inhibits appetite) resistance has been known to cause obesity and interestingly ER stress has been found to induce leptin resistance (Hosoi et al., 2008). Non-obese diabetic (NOD) mice exhibited dysfunction of the islet β-cell and glaring ER stress prior to onset of hyperglycemia in type 1 diabetes conditions. Several fold increase in NF-κB target genes indicated clear crosstalk between ER stress, cytokine signaling, and inflammation defining the loss of β-cells and their functions in NOD mice (Tersey et al., 2012). Pre-existing ER stress though mild, sensitizes the pancreatic cells to weak inflammatory stimulus causing an aggravated inflammatory response and accelerating the development of type 1 diabetes. Miani et al. (2012) showed involvement of ER stress specifically IRE1α-XBP1 pathway in NF-κB activation and expression of its target genes via modulation of fork head box O1 (FoxO1) protein concluding that β-cell-ER stress triggers exacerbated local inflammation. ER-stressed smooth muscle cells locally boost leukocyte adhesion by forming a hyaluronan rich extracellular matrix promoting inflammatory responses (Majors et al., 2003). Paneth and Goblet cells of intestinal epithelium are XBP1-dependent for survival and to execute their secretory functions. Intestinal inflammation in inflammatory bowel disease (IBD) emerges from dysfunction in these cells due to excessive ER stress, which may be an after effect of broad range of factors like primary genetic (abnormalities in UPR, XBP1) and secondary (inflammation, environmental) (Kaser and Blumberg, 2009; Kaser et al., 2011). There exist some complex connections between ER stress and inflammatory responses, which are cell type or condition specific and vary with different metabolic conditions causing vivid increase in inflammatory mediators. All the three sensors of UPR pathway are capable of NF-κB activation (Hotamisligil, 2010) (Figure 3).

### IRE1α–NF-κB

ER stress-induced activation of IRE1α kinase activity recruits adaptor protein TNF receptor-associated factor 2 (TRAF2), c-Jun-N-terminal inhibitory kinase (JIK), Jun activation domain-binding protein-1 (JAB1), and ASK1-interacting protein 1 (AIP1) to initiate downstream signaling pathways. IRE1α decides the cellular fate, i.e., survival via UPR or death via apoptosis; regulatory mechanisms of which are not yet entirely understood. JAB1 was suggested to be a decisive molecule: interacting with IRE1α under weak ER stress stimulation to suppress UPR and dissociating to allow IRE1α activation and the UPR under strong ER stressed conditions (Oono et al., 2004). Activated IRE1α recruits adaptor protein TRAF2, and c-JNK activation is followed. Dominant negative TRAF2 with a truncated N-terminal RING effector domain inhibited JNK activation via IRE1α (Urano et al., 2000). In response to ER stress-inducing agents like thapsigargin and tunicamycin, IRE1α–TRAF2 pathway is known to induce NF-κB activation, which is inhibited by both dominant negative IRE1α and TRAF2 (Leonardi et al., 2002; Kaneko et al., 2003). Hu et al. (2006) reported that the ER stress inducers induce IRE1α to form a complex with IKK essentially through TRAF2, thereby activating NF-κB by promoting degradation of IκBα resulting in NF-κB nuclear translocation. This was accompanied by upregulation of TNFα (but not FasL and TRAIL) in an IRE1α- and NF-κB-dependent manner (not via TNFR1 signaling) and downregulation of TRAF2, which weaken TNFα-induced activation of NF-κB and JNK, and triggered TNFα-induced apoptosis. JIK binds to both TRAF2 and IRE1α, causing increase in JNK activation under ER stress, thus modulating IRE1α–TRAF2 signaling events. TRAF2 also interacts with procaspase-12 under ER stress thereby promoting clustering and cleavage to its active caspase-12 form and initiating apoptotic signaling pathways (Yoneda et al., 2001). Luo et al. (2008) using AIP1 knockout cells concluded that AIP1 is involved in ER stress-activated IRE1α–JNK/XBP1 pathway and that IRE1α–AIP1 complex is essential for subsequent recruitment of TRAF2 to IRE1α. JNK, member of the mitogen activated protein kinase (MAPK) superfamily, phosphorylates and activates several transcription factors such as c-Jun, ATF2, p53, c-Myc, and many apoptosis signaling proteins like Bcl-2, Bcl-xL, Bad, and Bim (Bogoyevitch and Kobe, 2006). Activated JNK induces the expression of inflammatory genes by phosphorylating the transcription factor activator protein 1 (AP1), which is a dimer of monomers from various protein families like JUN, FOS, ATF, and MAF which interact to form different combinations of AP-1 (Davis, 2000). The various genes that are regulated by AP-1 are combination specific and execute different biological functions. They induce inflammation via promoting transcription of genes for cytokines, chemokines, and other proinflammatory molecules (Angel et al., 2001; Shaulian and Karin, 2001; Eferl and Wagner, 2003).

Knockdown experiments unveiled that loss of either IRE1α or PERK curtailed NF-κB signaling in ER stress pathways, signifying combinatorial and synergistic effect of both sensors for complete NF-κB activation. IRE1 (kinase domain)-TRAF2–IKK signaling, not via JNK, leads to ER stress-induced NF-κB activation (Tam et al., 2012). Spliced XBP1 and ATF4, induce production of the inflammatory cytokines/chemokines IL-8, IL-6, and monocyte chemoattractant protein 1 (MCP1) in human aortic endothelial cells (Gargalovic et al., 2006). Malfunctioning of Paneth cells and heightened proinflammatory response in XBP1-deficient intestinal epithelial cells (IEC) provided evidence of XBP1 anomalies in IEC as being exclusively responsible for intestinal inflammation (Kaser et al., 2008).

### PERK–NF-κB

PERK branch of UPR can potentially induce NF-κB essentially by its eIF2α-mediated attenuation of translation; successively inhibiting the synthesis of IκBα. It was also found that half life of this inhibitor is less compared to NF-κB culminating in increased ratio of NF-κB to IκB, which facilitates NF-κB’s nuclear translocation to activate its target genes in response to ER stress (Deng et al., 2004). Comparable results were also obtained by Wu et al. (2004), which demonstrated that ultraviolet light inhibits new IκBα synthesis that can be reversed by expression of an eIF2α (S51A) mutant.

### ATF6–NF-κB

Subtilase cytotoxin from Shiga strain of *E. coli* is a serine protease, which enters the cell and makes a specific single site cleavage in GRP78 (Paton et al., 2006). This causes the GRP78 to dissociate from the ER stress sensors and activate an early ER stress response qualitatively similar to that observed with the certified UPR-inducing chemical agents (Wolfson et al., 2008). Yamazaki et al. (2009) made a significant contribution by dissecting the UPR pathways involved in subtilase triggered NF-κB activation. The initial breakdown of GRP78 provoked the UPR triggering transient Akt phosphorylation and subsequent NF-κB activation exclusively via ATF6 signaling; as dominant negative inhibition of IRE1α, XBP1, or PERK did not attenuate activation of NF-κB. Zhang et al. (2006) also claimed that under ER stress, CREBH and ATF6 may dimerize and synergistically activate transcription of major APR genes inducing a systemic inflammatory response.

## NF-κB Activation in Neurodegeneration

In the central nervous system, NF-κB complexes are expressed by neurons, glial cells, and oligodendrocytes. Constitutive NF-κB activity is fundamental to physiological processes of brain development, synaptic signaling that govern learning and memory, and neuroprotection. Nonetheless, inducible NF-κB activity is observed in pathological conditions like trauma, ischemia, neurodegenerative diseases, memory dysfunction, and many more. The existence of diverse NF-κB complexes (Rel/NF-κB homodimers and heterodimers), their specificities for certain cell types, activating stimuli, responsive genes, explains the contradictory function of NF-κB in cell death and survival (Bhakar et al., 2002; Pizzi and Spano, 2006). Stimulatory signals for NF-κB activation in neurons can be stress- or injury-related or on exposure to cytokines or other signaling molecules including TNFα, glutamate, NGF, ADNF, and sAPP. These are capable of triggering downstream kinase cascades like calcium/calmodulin-dependent kinase II, Akt, protein kinase C, which may terminate in high constitutive neuronal NF-κB activity leading to signal transduction pathways and expression of inflammatory cytokines, chemokines, immune receptors, and cell surface adhesion molecules (Pahl, 1999; Li and Stark, 2002). NF-κB induces genes supporting cell survival like those of inhibitors of apoptosis proteins (IAPs), BCL-2s, TRAF1/TRAF2, and SOD. Glucose deprivation induces injury and excitotoxicity stress elevates intracellular calcium concentrations in embryonic neurons to toxic levels, which on TNFα administration are decreased by regulating the expression of proteins involved in calcium homeostasis thereby promoting neuronal survival. TNFα also protects neurons by increasing expression of anti-apoptotic proteins Bcl-2 and Bcl-xL in hypoxic or nitric oxide-induced injury (Cheng et al., 1994; Mattson et al., 1997; Tamatani et al., 1999). Downregulation of NF-κB in the hippocampus is associated Aβ1–42-mediated decline of neurogenesis (Zheng et al., 2013).

In glial cells (microglia and astrocytes), inducible NF-kB activity may indirectly promote neuronal death by producing large amounts of NO, proinflammatory cytokines interleukin-1b (IL-1b), interleukin-6 (IL-6) and TNF-α, ROS, and excitotoxins (John et al., 2003; Kim and de Vellis, 2005; Hsiao et al., 2013). Thus activation of NF-κB in neurons could promote their survival, whereas activation of NF-κB in glial cells may induce the production of neurotoxins depending on the context, i.e., cell type, stimulus, duration, and threshold levels of effectors (Mattson and Meffert, 2006). Nonaka et al. (1999) demonstrated remarkable prolonged activation of NF-κB persistent up to a year in glia following brain trauma in experimental rats suggesting NF-κB’s role in long-term inflammatory processes.

### Therapeutic implications

Researchers have concentrated their focus on identifying small molecules acting as chaperones to help stabilize misfolded proteins facilitating protein folding and alleviating ER stress. 4-Phenyl butyric acid (4-PBA) and taurine-conjugated ursodeoxycholic acid (TUDCA) are being explored in this regard for various diseases like colitis, atherosclerosis, and type 2 diabetes (Ozcan et al., 2006; Erbay et al., 2009; Cao et al., 2013; Vang et al., 2014). It is also known that Lipopolysaccharides (LPS) can induce various ER stress markers such as GRP78, CHOP in the lung tissues of LPS-treated mice while an ER stress inhibitor 4-phenylbutyrate (PBA), can reduce the LPS-induced lung inflammation in mice model (Kim et al., 2013). A novel strategy of inhibiting IRE1α endonuclease activity, without affecting its kinase activity has come to light identifying small molecules like MK3946 and STF-083010, as therapeutic options for multiple myeloma (MM) therapy. MK3946, an IRE1α endoribonuclease domain inhibitor, blocked XBP1 splicing causing death of MM cells and/or enhanced sensitivity of MM cells to other ER stress-inducing drugs bortezomib and 17-AAG, thereby proving to be of efficient lead for MM treatment (Mimura et al., 2012). Dinaciclib (SCH727965), a potent inhibitor of cyclin-dependent kinase (CDKs) 1/2/5/9, at extremely low (e.g., nmol/L) concentrations downregulated thapsigargin and tunicamycin-induced XBP1s and Grp78 expression in human leukemia and myeloma cells in association with evident cell death. It also markedly reduced myeloma cell growth in *in vivo* models (Nguyen and Grant, 2014). Similarly, STF-083010 exhibited cytostatic and cytotoxic activity in various MM cell lines and also effectively reduced tumor grown as xenografts in NSG mice models. It showed significant *ex vivo* toxicity to CD138^+^ cells (marker for plasma and MM cells) isolated from MM patients (Papandreou et al., 2011). Another approach in targeting UPR for therapeutic applications found GSK2656157, an ATP-competitive inhibitor of PERK enzyme, which inhibited multiple human tumor xenografts growth in mice (Atkins et al., 2013). In diet-induced obesity (DIO), C57BL/6J mice, atorvastatin treatment effectively improved pancreatic β cell function through improved proliferation, sensitivity to glucose, and attenuated ER stress (Chen et al., 2014). Gemigliptin, a dipeptidyl peptidase-IV inhibitor efficiently inhibited ER-stress-mediated apoptosis and inflammation in cardiomyocytes via Akt/PERK/CHOP and IRE1α/JNK-p38 pathways proposing to have direct beneficial effects to prevent the progression of cardiovascular diseases (Hwang et al., 2014). In Sprague-Dawley rats, post traumatic brain surgery administration of docosahexaenoic reduced ER stress marker proteins, ubiquitinated proteins, amyloid precursor protein/p-Tau proteins, and neurological deficits demanding further research (Begum et al., 2014). Wang et al. (2014) showed that Propofol, a clinically used anesthetic agent up regulates BiP, XBP1s, and cleaved ATF6, which may be involved in the adaptive ER stress and attenuates ER stress-induced phosphorylation of PERK and eIF2a, and inhibits the up-regulation of ATF4 and CHOP as well. Synthetic compound libraries targeting specific components of ER stress pathways are being evaluated in *in vitro* and *in vivo* conditions. For instance chromenone-based inhibitors of IRE1 RNase activity and small-molecule PERK inhibitors have been tested in cell lines (Pytel et al., 2014; Ranatunga et al., 2014). Interleukin-1 receptor-associated kinase-2 (IRAK2) is recently being identified as amplifier of IRE pathway in addition to its known functions in innate immunity. Therapeutic targeting of this molecule shall shed some light in understanding of pathogenesis of ER stress-related and inflammatory diseases (Benosman et al., 2013).

## Conclusion

Endoplasmic reticulum is a dynamic organelle orchestrating several crucial pathways that decide cellular fate. It senses alterations in ER homeostasis and triggers UPR pathways with an aim to initially restore homeostasis by activating genes involved in protein folding and degrading machinery transcribed by factors like ATF6, XBP1, and ATF4. If unresolved, it initiates cell death pathways. Under such situations, UPR pathways triggers, affects, and integrates with mitochondrial cellular signaling pathways. Neurodegenerative diseases, inflammatory diseases, cardiovascular diseases, diabetes mellitus, cancer, and several metabolic diseases have perturbed ER functions, which contribute to their pathogenesis to some extent considering the existence of other complications like inflammation or oxidative stress. Recent findings have revealed interconnections between ER stress, inflammation, and oxidative stress pathways under pathological conditions. Thus, further understanding of the molecular mechanisms in these interconnecting pathways occurring in numerous diseases may lead to discovery of novel therapeutic targets.

However, comprehensive research is demanded to aim UPR in diseases, wherein side effects, efficacy, and safety are major concerns. Nonetheless, there still exists a dilemma regarding ER stress being a cause or consequence for a particular diseased condition. Mitigating ER stress will certainly be of therapeutic significance keeping in mind that components involved in ER stress-induced inflammation are targeted specifically with minimal side effects and only desired cells are acted upon sparing the healthy cells. Focused research and in-depth investigations in this direction are needed for a new therapeutic strategy.

## Conflict of Interest Statement

The authors declare that the research was conducted in the absence of any commercial or financial relationships that could be construed as a potential conflict of interest.

## Abbreviations

ADNF, activity-dependent neurotrophic factor; AIP1, ASK1-interacting protein-1; ALS, amyotrophic lateral sclerosis; AMPA, α-amino-3-hydroxy-5-methylisoxazole-4-propionate acid; AP-1, activator protein-1; APR, acute phase response; ARD, ankyrin repeat domain; ASK1, apoptosis signal-regulating kinase; ATF4, activating transcription factor 4; ATF6, activating transcription factor 6; ATP, adenosine 5-triphosphate; Bad, Bcl-2-associated death promoter; BAFF, B cell activating factor; BAPTA-AM, 1,2-Bis(2-aminophenoxy)ethane-*N*,*N*,*N*′,*N*′-tetraacetic acid tetrakis(acetoxymethyl)ester; Bcl2, B-cell lymphoma 2; Bcl xl, B-cell lymphoma-extra large; BHA, butylated hydroxyanisole; Bim, B-cell lymphoma 2 interacting mediator of cell death; BIP, immunoglobulin heavy chain binding protein; CDDO, 2-cyano-3,12-dioxoolean-1,9-dien-28-oic acid; CDDO Me, methyl 2-cyano-3,12-dioxooleana-1,9(11)dien-28-oate, Bardoxolone methyl; CEMB, cyano enone of methyl boswellates; CHOP, CCAAT/enhancer-binding protein homologous protein; CNX, Calnexin; CREB, cAMP response-element-binding; CRP, C-reactive protein; CRT, Calreticulin; DTT, dithiothreitol; EDEM, ER degradation-enhancing α-mannosidase-like protein; eIF2α, eukaryotic translation initiation factor; ER, endoplasmic reticulum; ERAD, ER-associated degradation; ERKs, extracellular signal-regulated kinases; ERO, ER membrane associated oxidoreductin; ERSE, ER stress response element; ERQC, Endoplasmic reticulum quality control system; FAD, Flavin adenine dinucleotide; FoxO1, fork head box O1; GLS, Golgi-localization signal; GRP, glucose-regulated protein; GSH, glutathione; GSSH, glutathione to oxidized glutathione; IBD, inflammatory bowel disease; IEC, intestinal epithelial cells; IKK, IκB kinase; IAPs, inhibitor of apoptotic proteins; IL-1R, interleukin 1-receptor; InsP3, inositol 1,4,5-triphosphate RyRs, ryanodine receptors; IRAK2, interleukin-1 receptor-associated kinase-2; IRE1α, inositol-requiring kinase 1; IRES, internal ribosome entry site; JAB1, Jun activation domain-binding protein-1; JIK, c-Jun-N-terminal inhibitory kinase; LPS, lipopolysaccharides; MAM, mitochondrial associated membranes; MAPK, mitogen activated protein kinase; MAPKKK, mitogen activated protein kinase kinase kinase; MCP1, monocyte chemoattractant protein-1; MM, multiple myeloma; NAC, *N*-acetyl-l-cysteine; nACh, nicotinic acetylcholine; NDGA, nordihydroguaiaretic acid; NEMO, NF-kappa-B essential modulator; NES, nuclear export sequence; NF-κB, nuclear factor κB; NGF, nerve growth factor; NIK, NF-κB-inducing kinase; NLS, nuclear localization signal; NMDA, *N*-methyl-d-Aspartate; NO, nitric oxide; NOD, non-obese diabetic; Nrf2, nuclear erythroid 2 p45-related factor 2; Oasis, old astrocyte specifically induced substance; PBA, 4-phenylbutyrate; PDI, protein disulfide isomerase; PDTC, pyrrolidine dithiocarbamate; PERK, double-stranded RNA-activated protein kinase (PKR)-like endoplasmic reticulum kinase; RER, rough endoplasmic reticulum; RHR, Rel homology region; RIP, regulated intramembrane proteolysis; RNS, reactive nitrogen species; ROI, reactive oxygen intermediates; ROS, reactive oxygen species; S1P, site-1 protease; S2P, site-2 protease; SAP, serum amyloid P-component; sAPP, secreted β-amyloid precursor protein; SER, smooth endoplasmic reticulum; SERCA, Sarco/ER calcium-ATPase; SOD, superoxide dismutase; SRP, signal recognition particle; sXBP1, spliced XBP1; TAD, transcription activation domain; TCA, tricarboxylic acid; TLR, Toll-like receptor 4; TMB-8, 3,4,5-trimethoxybenzoic acid 8-(diethylamino)octyl ester; TNFR, tumor necrosis factor receptor; TRAF2, tumor necrosis factor receptor-associated factor 2; TUDCA, taurine-conjugated ursodeoxycholic acid; UPR, unfolded-protein response; XBP1, X-box-binding protein 1.
